# Activity of ipilimumab plus nivolumab in avelumab-refractory Merkel cell carcinoma

**DOI:** 10.1007/s00262-020-02832-0

**Published:** 2021-01-13

**Authors:** Valerie Glutsch, Hermann Kneitz, Anja Gesierich, Matthias Goebeler, Sebastian Haferkamp, Jürgen C. Becker, Selma Ugurel, Bastian Schilling

**Affiliations:** 1 Department of Dermatology, Venereology and Allergology, University Hospital Würzburg, Josef-Schneider-Str. 2, 97080 Würzburg, Germany; 2 Department of Dermatology, University Medical Center, Regensburg, Germany; 3 Deutsches Konsortium Für Translationale Krebsforschung (DKTK), Translational Skin Cancer Research, Essen and Heidelberg, Germany; 4 Department of Dermatology, University Hospital, University Duisburg-Essen, Essen, Germany

**Keywords:** Merkel cell carcinoma, Resistance, Avelumab, Ipilimumab, Nivolumab

## Abstract

**Background:**

Merkel cell carcinoma (MCC) is a rare and aggressive neuroendocrine cutaneous malignancy with poor prognosis. In Europe, approved systemic therapies are limited to the PD-L1 inhibitor avelumab. For avelumab-refractory patients, efficient and safe treatment options are lacking.

**Methods:**

At three different sites in Germany, clinical and molecular data of patients with metastatic MCC being refractory to the PD-L1 inhibitor avelumab and who were later on treated with combined IPI/NIVO were retrospectively collected and evaluated.

**Results:**

Five patients treated at three different academic sites in Germany were enrolled. Three out of five patients investigated for this report responded to combined IPI/NIVO according to RECIST 1.1. Combined immunotherapy was well tolerated without any grade II or III immune-related adverse events. Two out of three responders to IPI/NIVO received platinum-based chemotherapy in between avelumab and combined immunotherapy.

**Conclusion:**

In this small retrospective study, we observed a high response rate and durable responses to subsequent combined immunotherapy with IPI/NIVO in avelumab-refractory metastatic MCC patients. In conclusion, our data suggest a promising activity of second- or third-line PD-1- plus CTLA-4-blockade in patients with anti-PD-L1-refractory MCC.

## Introduction

Merkel cell carcinoma (MCC) is a highly aggressive and rare cutaneous malignancy that is induced by the Merkel cell polyomavirus (MCPyV) or ultraviolet irradiation [1]. Until recently, treatment of advanced or metastatic MCC was limited to chemotherapy showing significant but short-lived activity [2].

Immune checkpoint blockade (ICB) has shown high response rates in metastatic MCC [3–5]. In the first-line setting, PD-1 inhibition with pembrolizumab or PD-L1 inhibition with avelumab results in high objective response rates of 56% and 62.1% [4–6]. To date, the PD-L1 inhibitor avelumab is the only approved treatment for advanced MCC [6]. However, primary and acquired resistance to avelumab remains a so far unsolved clinical challenge.

Unfortunately, only limited and heterogeneous data are existing on metastatic MCC patients being refractory to PD-1- or PD-L1-blockade. LoPiccolo et al. presented a case series with 31% (4/13) patients responding to IPI/NIVO or ipilimumab monotherapy after progression on anti-PD-1 monotherapy or anti-PD-1 containing experimental regimes [7]. In this case series, a single MCC patient with primary resistance to palliative avelumab exposed to second-line IPI/NIVO was included. With further approved treatment options being limited to chemotherapy inducing only transient responses [8], investigation of these patients regarding subsequent ICB seems vitally important.

Here, we report a retrospective multicenter cohort of five patients with metastatic MCC and primary resistance to avelumab being treated with IPI/NIVO. Our data support our initial observation [9] that combined ICB is safe and active in avelumab-refractory MCC.

## Patients and methods

### Patient data

**Fig. 1:**
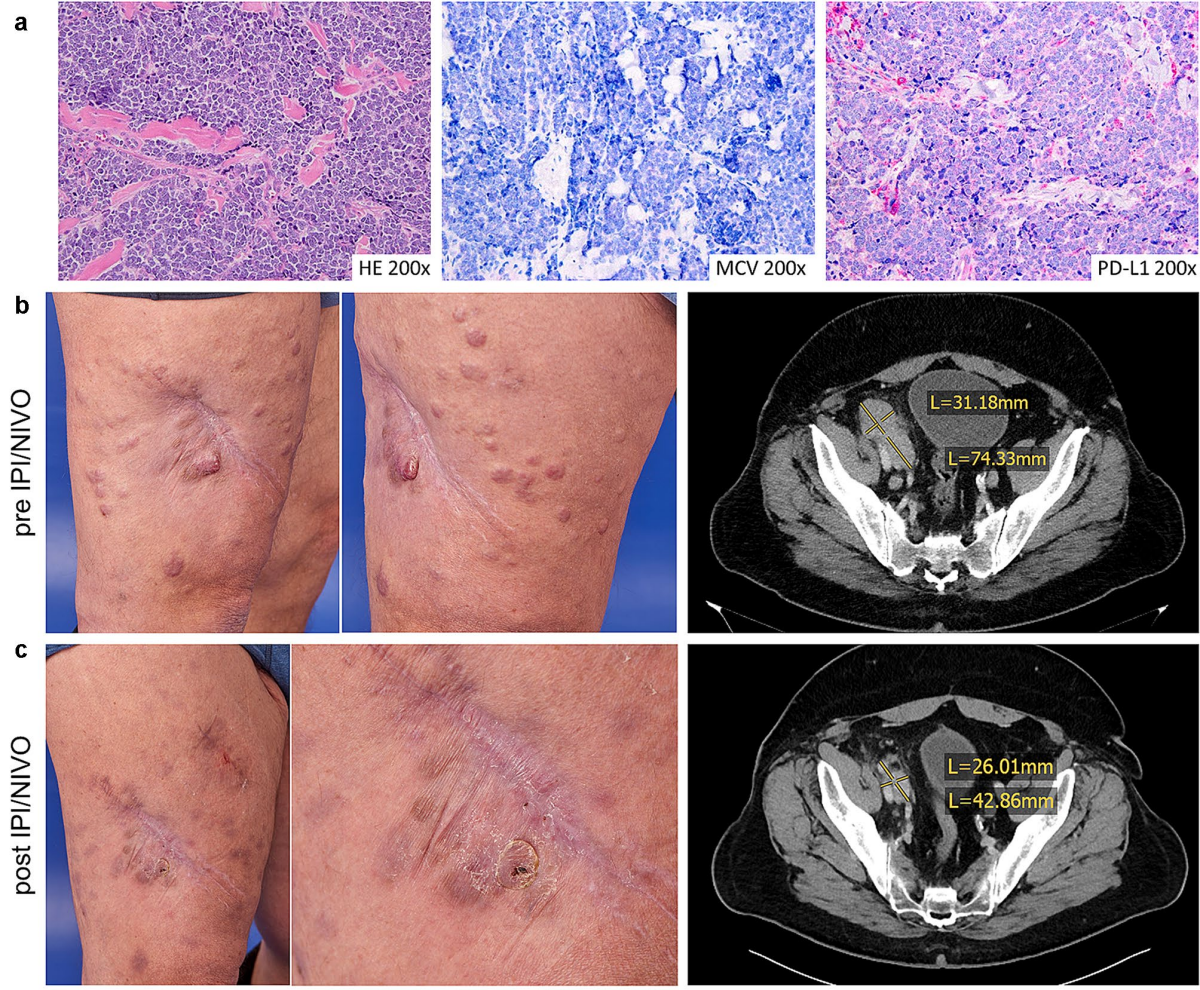
Representative photos, CT scans and immunohistochemistry of patient 3. **a** Hematoxylin and eosin (H&E) staining as well as immunochemistry for MCPyV and PD-L1 (clone 22–8) of tissue obtained prior to initiating IPI/NIVO after progression upon chemotherapy (cutaneous metastasis, right thigh). **b** Clinical presentation before initiating IPI/NIVO and after 4 cycles of IPI/NIVO (right thigh). **c** CT scans before IPI/NIVO and after 4 cycles of IPI/NIVO showing a partial remission of a parailiacal lymph node metastasis

Clinical and molecular data of consecutive patients with metastatic MCC who had been refractory to the PD-L1 inhibitor avelumab and were later on treated with IPI/NIVO were retrospectively collected at three academic sites in Germany. Data were obtained from hospital records by chart review. Tissue used was collected during routine care for diagnostic or therapeutic reasons. Due to the retrospective nature of the study and the collection of anonymous patient data, informed consent was waived by the Ethics Committee of the University Hospital Würzburg. One of the patients was reported previously and was included with additional follow-up [9]. Progression-free survival (PFS) was calculated from the first course of IPI/NIVO to the last tumor assessment. Overall survival (OS) was calculated from the first course of IPI/NIVO to the last consultation, respectively, the date of death (Fig. 1).

### Immunohistochemistry

Immunohistochemistry for PD-L1 and MCPyV was performed as described [9].

## Results

### Patient demographics

Five patients, 80% (4/5) being male, with metastatic MCC stage IV (UICC 2017) were included in our analysis. The age at first MCC diagnosis ranged from 57 to 70 years. Patient demographics and outcome are summarized in Table 1.

**Table 1:** Patient characteristics and treatment outcomes

Patient	1	2	3	4	5
Age (years) at first diagnosis	57	70	67	64	58
Sex	Male	Male	Male	Male	Female
Stage (UICC 2017)	IV	IV	IV	IV	IV
MCPyV	Positive	Positive	Negative	Negative	Positive
PD-L1	Negative	Negative	Negative	na	Negative
Avelumab					
Number of courses	7	9	2	4	4
BOR (RECIST 1–1)	PD	SD	PD	PD	PD
irAE	–	PNP°II	Pneumonitis°II, Hepatitis °II	–	–
Subsequent therapies					
Therapy 1	–	–	C + E*	C + E** + radiotherapy	C + E*
BOR (RECIST 1–1)	–	–	PD	PD	PR
Therapy 2	–	–	–	Nivolumab	Avelumab
BOR (RECIST 1–1)	–	–	–	SD/PD	PD
IPI/NIVO					
LDH	Elevated	Elevated	Elevated	Normal	Elevated
CRP	1.16 mg/dl (ULN < 0.5)	0.9 mg/dl (ULN < 0.5)	0.52 mg/dl (ULN < 0.5)	1.1 mg/dl (ULN < 0.5)	156 mg/l (ULN < 3)
ECOG PS	0	0	1	1	2
Dosing	IPI1/NIVO3	IPI1/NIVO3	IPI1/NIVO3	IPI3/NIVO1	IPI3/NIVO1
Number of courses	4	1	4	4	2
BOR (RECIST 1.1)	CR	PD	PR	PR	PD
irAE	Fatigue °I	–	–	–	–
Maintenance therapy (NIVO)	No	No	No	Yes	No
PFS (months)	12.2	0.5	> 3.3	> 1.7	0.9
OS (months)	> 15.9	1.1	> 4.0	> 3.4	1.4
Progressed?	Yes	na	No	No	na
Alive?	Yes	no	Yes	Yes	No

*MCC* Merkel cell carcinoma; *UICC* Union international contre le cancer; *MCPyV* Merkel cell polyomavirus; *BOR* best overall response; *irAE* immune-related adverse event; *LDH* Lactate dehydrogenase; *CRP* C-reactive protein; *ECOG PS* Eastern Cooperative Oncology Group Performance Status; *PFS* progression-free survival; *OS* overall survival; *na* not applicable; *PD* progressive disease; *SD* stable disease; *PR* partial remission; *CR* complete response *PNP* peripheral polyneuropathy; *IPI1/NIVO3* ipilimumab 1 mg per kg + nivolumab 3 mg per kg; *IPI3/NIVO1* ipilimumab 3 mg per kg + nivolumab 1 mg per kg

^*^Carboplatin + etoposide

^**^Cisplatin + etoposide

### First-line treatment with avelumab

All five patients received the PD-L1 inhibitor avelumab (10 mg per kilogram of body weight) as first-line systemic treatment for metastatic disease. The number of courses ranged from 2 to 9. Four patients showed progressive disease (PD) in the first tumor assessment after therapy initiation while one patient showed short-lived stabilization for 6.4 months (stable disease (SD) according to RECIST 1.1) followed by disease progression. Treatment-related immune-related adverse events (irAE) of grade II or III (according to Common Toxicity Criteria of Adverse Events, CTCAE 4.03) were observed in two of five patients. One patient developed peripheral polyneuropathy which improved upon intravenous methylprednisolone to grade I. Another patient developed pneumonitis grade II and hepatitis grade III, and was treated with methylprednisolone. Pneumonitis improved to grade I and hepatitis resolved.

### Subsequent treatment regimes

Two patients had surgery or surgery plus radiotherapy after having progressed under avelumab. Patient three received three courses of chemotherapy with carboplatin plus etoposide and showed PD (RECIST 1.1) in the first tumor assessment. The two remaining patients had two systemic treatment regimes in between avelumab and IPI/NIVO. Patient four progressed after five courses of radiochemotherapy with cisplatin plus etoposide and received PD-1 blockade with nivolumab subsequently. Tumor assessment (RECIST 1.1) showed PD with progressive metastases after four courses of nivolumab. Patient five received six courses of chemotherapy with carboplatin plus etoposide and showed a partial remission (PR, RECIST 1.1) after 3 months of treatment. Unfortunately, she progressed 2 months later and was re-exposed to avelumab showing PD after three courses.

### Ipilimumab plus nivolumab

All five patients received combined IPI/NIVO (three patients with IPI 1 mg per kilogram plus NIVO 3 mg per kilogram; two patients with IPI 3 mg per kilogram plus NIVO 1 mg per kilogram). Three patients underwent four courses of IPI/NIVO, while the other two patients received only two courses, respectively, one course of IPI/NIVO due to early tumor progression. Three out of five patients investigated responded to combined IPI/NIVO according to RECIST 1.1 (overall response rate (ORR) 60%). Among these three patients, two had at least one additional systemic therapy in between avelumab and IPI/NIVO. In the patient showing a complete remission (CR) with no sign of residual disease after four courses of IPI/NIVO, a maintenance therapy with nivolumab was omitted. One patient showing a PR after four courses of IPI/NIVO is receiving maintenance therapy with nivolumab, whereas we refrained from maintenance therapy in patient three due to a deep PR. Combined ICB was tolerated well. There were no irAE apart from a fatigue grade I.

### Follow-up

Patients three and four have not relapsed until now with follow-up being 3.4, respectively, 4.0 months. The patient with a CR after IPI/NIVO did not receive maintenance therapy and relapsed after 12.2 months. Two patients did not respond to combined ICB and died after one, respectively, two courses of IPI/NIVO due to tumor progression.

## Discussion

The activity of ICB in MCC has revolutionized treatment and in contrast to chemotherapy durable tumor regression can now be observed [4–6, 8]. Although the cell of origin remains elusive, MCC shows an extraordinary biology with ~ 80% of tumors being associated with the insertion of MCPyV into the tumor genome and ~ 20% being linked to the exposure to ultraviolet (UV) light [10, 11]. Both might explain the high response rate to ICB. Consequently, MCC is a tumor entity with characteristics providing an auspicious rationale for response to ICB.

In contrast to MCPyV-positive tumors, most of the virus-negative, presumably UV-induced MCCs present with a strikingly high tumor mutational burden (TMB) [11, 12]. A high TMB is already known as a marker for response to ICB in other tumor entities [13]. Topalian et al. recently reported that MCC patients with higher TMB did not show superior clinical benefit when receiving neoadjuvant PD-1 blockade with nivolumab [14]. For second-line avelumab, a weak association of OS and PFS with a higher TMB was found [15]. TMB might be a rather predictive biomarker for a specific ICB in a given entity instead of a universal indicator of clinical benefit from immunotherapy [16]. Therefore, TMB was not analyzed in our cohort and it remains controversial if TMB is of relevance in MCC. Apart from TMB, the presence of viral antigens has been proposed recently to explain responses to ICB in virus-positive tumors with comparable low TMB [5]. However, responses to PD-1 blockade with pembrolizumab in the first-line setting have been observed in both MCPyV-negative and -positive tumors [5]. For second-line avelumab and neoadjuvant nivolumab, no association of MCPyV status and response was reported [3, 14, 15]. Our data support the notion that the presence of the MCPyV is not associated with benefit from ICB since we observed responses to IPI/NIVO in virus-negative tumors. Taken together, surrogate markers indicating immunogenicity of cancer cells are present in MCC. However, their predictive value and clinical usefulness to foresee clinical benefit from ICB in MCC remain unclear and warrant further investigation [4, 5].

Unfortunately, treatment options for ICB refractory metastatic MCC patients are limited and only few data exist on subsequent therapies in PD-1- or PD-L1-resistant patients. In this perspective, primary and acquired resistances have to be distinguished. Based on the reported duration of response, primary resistance seems to be the more important clinical issue in MCC [15]. Even though PD-1 and PD-L1 are known to impact the same immunoregulatory pathways, patients having progressed after blockade of one might benefit from subsequent therapy with the other. In theory, anti-PD-1 antibodies might also block all unknown ligands of PD-1 while anti-PD-L1 antibodies might block all unknown receptors of PD-L1. So far, a single case of a response to avelumab after PD under anti-PD-1 monotherapy with pembrolizumab and subsequent IPI/NIVO was published [7]. In the same report, PD-1-based therapies in MCC patients after treatment with avelumab-based therapies are described. Within this very heterogenous patient cohort, five patients received avelumab as monotherapy (*n* = 2), as adjuvant therapy (*n* = 1) or as part of a combinatory regime (*n* = 2). Of those who received palliative avelumab as monotherapy, one patient showed an initial PR while the other one was primary resistant. Both patients did not show an objective response to subsequent IPI/NIVO. To avoid such a heterogeneity making interpretation difficult, databases at participating sites were searched only for MCC patients who received palliative avelumab monotherapy. Based on findings in MCC patients treated with an adaptive T cell therapy and ICB, acquired resistance to ICB seems to be determined by genetic events that cannot be reversed by switching to a different ICB [17]. Thus, our analysis was restricted to patients with primary resistance to avelumab. Of note, two of our patients who responded to IPI/NIVO had chemotherapy or radiochemotherapy and anti-PD-1 monotherapy in between avelumab and IPI/NIVO. There are prospective data about responses to ICB in chemotherapy-refractory MCC patients [3]. In fact, a chemotherapy-induced sensitization of tumor cells to the patient’s immune response triggered by subsequent ICB seems possible. Data supporting this hypothesis are based on clinical and experimental evidence that defects in the DNA repair machinery might play a decisive role in the activity of ICB [18]. Since adding the CTLA-4 inhibitor ipilimumab has already shown anti-tumor activity in PD-1-refractory patients with metastatic MCC [7], combined ICB according to the ongoing CheckMate-358 study (NCT02488759) seems to be a promising treatment option for avelumab-refractory patients. Although careful patient selection makes interpretation of our case series reporting a high ORR of 60% after sequential administration of avelumab and IPI/NIVO easier, a prospective clinical trial is needed to fully evaluate this intervention.

Combined IPI/NIVO is known for its high toxicity [19], nevertheless it was tolerated surprisingly well in our cohort without the occurrence of any grade II or III AE so far. When combining IPI/NIVO, toxicity seems to be dependent on the dosing of ipilimumab [20]. In two patients having received four courses of IPI 1 mg per kilogram plus NIVO 3 mg per kilogram (dosing chosen according to the ongoing CheckMate-358 study) and one patient having received four courses of IPI 3 mg per kilogram plus NIVO 1 mg per kilogram only a fatigue grade I occurred. The two deaths were caused by tumor progression without any signs for irAE. Since three out of five patients received chemotherapy in between avelumab and combined ICB, an immunological exhaustion possibly minimized immune-related side effects. However, the low incidence of irAE might also be, at least in part, explained by the fact that treatment was performed outside a clinical trial.

The major limitations of our report are the small number of patients and a short follow-up. Given the fact that metastatic MCC is a rare condition affecting elderly and fragile patients, we are confident to provide meaningful data. We can only provide data on the durability of the observed responses for one patient (PFS 12.2 months). Two of the three responses are in MCPyV-negative patients and ongoing at the time of our analysis, though with quite short follow-up. Therefore, additional experience with longer follow-up is needed.

In conclusion, our retrospective multicenter analysis provides data on the activity of combined IPI/NIVO in anti-PD-L1-refractory MCC patients. With responses in 3/5 patients including patients who received other therapies prior to IPI/NIVO, our data provide a rationale to offer combined ICB to patients with advanced MCC. Nevertheless, our results warrant further investigations and validation with larger cohorts and longer follow-up, ideally in a prospective clinical trial.

## Data Availability

Data will be provided upon request for reasonable academic studies by the corresponding author.
